# Spatial epidemiologic analysis of the liver cancer and gallbladder cancer incidence and its determinants in South Korea

**DOI:** 10.1186/s12889-021-12184-8

**Published:** 2021-11-14

**Authors:** Jieun Jang, Dae-Sung Yoo, Byung Chul Chun

**Affiliations:** 1 Department of Preventive Medicine, Korea University College of Medicine, 73, Goryeodae-ro, Seongbuk-gu, Seoul, 02841 Republic of Korea; 2 Veterinary Epidemiology Division, Animal and Plant Quarantine Agency, 177, Hyeoksin 8-ro, Gimcheon-si, 39660 Gyeongsangbuk-do Republic of Korea

**Keywords:** Spatial analysis, Disease hotspot, Liver neoplasms, Gallbladder neoplasms, Incidence, Korea, Bayesian analysis

## Abstract

**Background:**

There have been reports on regional variation in prevalence of hepatitis B and *C*, and *Clonorchis sinensis* (*C. sinensis*) infection, which indicates potential of spatial variation in liver cancer and gallbladder cancer incidence in Korea. Therefore, we aimed to assess the regional variation of liver and gallbladder cancer incidence and its determinants based on the regional distribution of risk factors, including hepatitis B infection in Korea.

**Methods:**

This study used an ecological study design and district-level cancer incidence statistics generated by the National Cancer Center. Spatial clusters of liver and gallbladder cancer incidence were detected based on spatial scan statistics using SaTScan™ software. We set the size of maximum spatial scanning window of 25 and 35% of the population at risk for analyses of liver and gallbladder cancer, respectively. Significance level of 0.05 was used to reject the null hypothesis of no cluster. We fitted the Besag-York-Mollie model using integrated nested Laplace approximations to assess factors that influence the regional variation in cancer incidence.

**Results:**

Spatial clusters with high liver cancer incidence rates were detected in the southwestern and southeastern regions of Korea. High gallbladder cancer incidence rates are clustered in the southeastern region. Regional liver cancer incidence can be accounted for the prevalence of high household income (coefficient, − 0.10; 95% credible interval [CI], − 0.18 to − 0.02), marital status (coefficient, − 0.14; 95% CI, − 0.25 to − 0.03), the incidence of hepatitis B (coefficient, 0.87; 95% CI, 0.29 to 1.44), and liver cancer screening (coefficient, 0.06; 95% CI, 0.00 to 0.12), while gallbladder cancer incidence was related to the prevalence of high household income (coefficient, − 0.03; 95% CI, − 0.05 to 0.00) and living close to a river with a high prevalence of liver fluke infection (coefficient, 0.55; 95% CI, 0.14 to 0.96).

**Conclusions:**

This study demonstrated geographic variation in liver and gallbladder cancer incidence, which can be explained by determinants such as hepatitis B, income, marital status, and living near a river.

**Supplementary Information:**

The online version contains supplementary material available at 10.1186/s12889-021-12184-8.

## Background

In 2017, liver cancer was still the sixth most commonly diagnosed cancer type in Korea (age-standardized incidence rates [ASR]: 26.8 and 7.2 per 100,000 for men and women, respectively), despite a continuous decreasing trend in its incidence rate from 1999 to 2017 [1]. Gallbladder cancer is a relatively rare type of cancer compared to other major cancer types in Korea (ASR: 8.0 and 5.5 per 100,000 for men and women, respectively); however, there was no significant decrease in the incidence rate from 1999 to 2017, and approximately 7000 people in Korea are still diagnosed with gallbladder cancer annually, of which 5000 people die.

Hepatitis B and C viruses and *Clonorchis sinensis* (*C. sinensis*), the Chinese liver fluke, are well-known risk factors for liver cancer supported by sufficient evidence [2, 3]. Additionally, clonorchiasis, an infectious disease caused by *C. sinensis*, has also reportedly been associated with the development of gallbladder stones [4] which has been suggested as a risk factor for gallbladder cancer [5, 6].

In Korea, there have been reports of regional variations in the prevalence of hepatitis B and *C. sinensis.* The prevalence of positive hepatitis B test results in Jeju Island, Gwangju, Busan, and the South Jeolla, and South Gyeongsang provinces was significantly higher than that of other regions [7]. Another study also reported a higher prevalence of *C. sinensis* infections in five major river basins in Korea [8]. Additionally, a higher prevalence of liver fluke infection was observed among gastrointestinal disease patients whose residence or place of birth was near rivers located in the Gyeongsang and South Jeolla provinces than among patients who lived or were born in regions near rivers located in other divisions [9]. These results imply that regional variation has the potential to cause a difference in liver and gallbladder cancer incidence rates in Korea due to regional differences in the prevalence of hepatitis B and *C. sinensis*.

In 2016, statistics on the incidence of 24 types of cancer in the Sigungu district unit, a second-level administrative district in South Korea, were released by the National Cancer Center for the first time [10]. A high incidence of liver cancer in southern regions including the South Gyeongsang and South Jeolla provinces and a high incidence of gallbladder cancer in the Gyeongsang province were observed in the reported statistics. However, it has not yet been determined whether the regions with high liver and gallbladder cancer incidence rates are a significant disease cluster which is an uncommon occurrence of medical conditions within a particular geographic location.

The application of spatial analysis in the medical field is diverse, such as evaluating accessibility to medical resources [11], exploring spatial pattern of disease or searching for spatial clusters of the diseases aforementioned, and identifying factors that can explain disease clusters and regional variations in diseases [12, 13]. It provides a basis for health policymakers to determine priorities and distribute healthcare resources and decide intervention for disease prevention and control [14]. In cancer research field, studies have been performed to evaluate the association between environmental factors and cancer incidence based on spatial analysis [15, 16]. These studies have reported the vulnerable areas for cancer incidence and suggested environmental factors that can be considered to resolve it by showing the inequality of cancer incidence and suggesting factors that can explain this imbalance. To date, it has not been evaluated that there are spatial clusters (hot spots) of liver and gallbladder cancer, which are regions where the incidence of disease is significantly higher than that of other regions, or whether regional factors can account for regional variations in liver and gallbladder cancer incidence in Korea.

Therefore, we performed this study to identify significant spatial clusters of liver cancer and gallbladder cancer incidence from 1999 to 2013 in Korea. Furthermore, we explored factors that could explain the spatial variations in liver and gallbladder cancer incidence.

## Methods

### Study design and data preparation

This study was based on an ecological study using district-level cancer incidence statistics released by the National Cancer Center and the regional characteristics of each Sigungu district.

In 2016, the National Cancer Center reported the Sigungu district-level incidence rates of 24 cancer types including most common cancers such as stomach, colon and rectum, lung, thyroid, breast, and liver from 1999 to 2013. They provided crude and age-standardized cancer incidence rates from three time periods (1999–2003, 2004–2008, and 2009–2013). We selected regional liver and gallbladder cancer incidence data from the Sigungu district-level caner incidence data above and used all 741 observations (247 Sigungu district level cancer incidence rates X 3 preiods) without selecting a regional cancer incidence rate of specific district. Liver and gallbladder cancers were defined as cancer cases according to the International Classification of Diseases for Oncology 3rd edition of C22 and C23-C24, respectively. Gallbladder cancer includes malignancies in the gallbladder and other or unspecified parts of the biliary tract.

We defined administrative boundaries, including 247 Sigungu districts, in 2004 as standard boundaries for district units in this study and obtained the administrative boundary shapefile from the Statistical Geographic Information Service (https://sgis.kostat.go.kr/).

For the periods of 1999–2003 and 2009–2013, some Sigungu districts were combined or separated; therefore, their boundaries differed to those of Sigungu districts obtained in 2004. Thus, for areas that needed to be combined, we assigned the weighted mean of cancer incidence rates using the proportion of the number of residents in Sigungu areas that were combined into one area. For the Sigungu districts that were separated into smaller regions, we allocated the same cancer incidence rates in the Sigungu district before they were separated into the smaller districts.

We considered several covariates, including demographic factors, socioeconomic status, health behavior, and health care utilization to explain the regional variation in liver and gallbladder cancer incidence. Covariate information was obtained from the Korea Community Health Survey (KCHS) data, which are representative community-based data gathered for the purpose of promoting the planning, implementation, monitoring, and evaluation of community health and disease prevention programs in Korea [17]. Information on the covariates considered in this study is presented in Supplementary Table 1. We summarized the individual-level information of covariates into Sigungu-level information by calculating the proportion of categorical variables. Subjects included in the KCHS were selected using multistage sampling to estimate representative statistics of the overall Korean population. To achieve unbiased estimates of the proportion or mean of covariates, sampling components (strata and cluster) and appropriate sampling weight were considered in the analyses. The survey procedures using SAS software (version 9.4; SAS Institute, Cary, NC, USA), which was developed for complex survey data analysis, was used in this study.

Although *C. sinensis* infection is a substantial risk factor for liver and gallbladder cancer, information on the prevalence of *C. sinensis* in each district is not available. Thus, we used the minimum distance from the national rivers located in regions showing a high prevalence of *C. sinensis* infection at the centroid of each Sigungu district as a proxy variable for *C. sinensis* infection. Calculation of distance from the rivers to the centroid, as described above, was performed using ArcGIS version 9.3 software (ESRI, Redlands, CA, USA). Since we hypothesized that liver and gallbladder cancer incidence rates in regions near the river with high *C. sinensis* infection were different to those in other regions, we classified the distance between the river and centroid of each Sigungu as dichotomous variables (distance < 10 km vs. ≥10 km) rather than using continuous values.

### Spatial cluster analysis

A spatial cluster for liver and gallbladder cancer incidence during three periods (1999–2003, 2004–2008, and 2009–2013) was identified based on the spatial scan statistics using SaTScan software™ with a normal model [18]. Spatial clusters can be recognized by imposing a scanning window, which is a potential candidate cluster, on the map, and comparing the expected value inside the window to the observed value by calculating the likelihood ratio. The spatial cluster is explored by repetitively calculating the likelihood ratio of each scanning window according to changing the location and size of the window, and defining the window with the maximum likelihood ratio as the first spatial cluster. The same process is performed to find secondary clusters that do not spatially overlap with the most likely cluster. We used an elliptic scanning window rather than a circular scanning window to detect long and narrow clusters with different shapes and directions.

The window size has a significant influence on the location and size of the cluster. If the window is too small, an actual cluster that is larger than the window may not be detected or only a partial area within the cluster will be discovered. Conversely, if the window is too large, there is a possibility that a small but meaningful cluster will be missed. Therefore, we searched for spatial clusters by changing the maximum spatial cluster size from 10 to 50% in 5% increments and finding the best fitted model based on the lowest value of the sum of the log likelihood ratios with consideration of penalty term consisting of the population in the identified clusters and the number of clusters. Consequently, maximum spatial scanning window sizes of 25 and 35% of the population at risk for liver and gallbladder cancer were used in the analyses. Monte Carlo simulation method was used to deal with the multiple comparison problem in the cluster analysis and *p*-values were generated based on 999 simulations. We mapped age-standardized incidence rates and spatial clusters for liver cancer and gallbladder cancer using the open-source geographic information system software QGIS™ (version 3.10).

### Assessment of determinants on regional variation in liver and gallbladder cancer incidence

We explored determinants that can explain regional variability in liver and gallbladder cancer incidence based on the Bayesian hierarchical spatial regression model. Before constructing the spatial model, we selected covariates to be included in the model to take the possibility of multicollinearity into consideration. We chose covariates that have the potential to be associated with liver and gallbladder cancer incidence by evaluating the association of each covariate with liver and gallbladder cancer based on the univariable linear regression model and selecting covariates with *p*-values < 0.2. Then, we constructed a multivariable model including all the selected covariates and estimated the variance inflation factors of each covariate. Finally, we determined covariates to establish the spatial model by excluding variables with variance inflation values ≥5.

We used the Besag-York-Mollie model to find determinants that could explain regional variation in liver and gallbladder cancer incidence with consideration of autocorrelation among regional cancer incidence rates and characteristics. The association between the potential determinant and the regional incidence rate of liver and gallbladder cancer was assessed by estimating the coefficient and two-tailed 95% CIs with a significance level of 0.05.

The spatial model was specified as eq. (1) below:

1 $$ {y}_i\sim Normal\left({\mu}_i,{\sigma}_i\right)\ {\mu}_i=\alpha +\sum \limits_{k=1}^K{\beta}_{ik}{x}_i+{\nu}_i+{\upsilon}_i $$

*·μ_i:linear prediction term;*

*·α:intercept;*

*·β_ik:coefficient for each potential determinant;*

*·ν_i:spatially unstructured random effect component;*

*·υ_i:spatially structured component*

*·i:each sigungu district number;*

*·k:each covariate number included in the model*

Among parameters in the model, coefficient for each potential determinant (*β*_*ik*_) indicates correlation between regional prevalence of characteristics and regional incidence of liver and gallbladder cancer. For example, the coefficient value of 1 indicates 1% increase in regional prevalence of certain characteristic is associated with increase in 1 cancer case per 100,000 persons.

To reduce the computational burden of Bayesian inference based on the Markov chain Monte Carlo methods, we performed Bayesian inference for the spatial model using integrated nested Laplace approximation. We assigned a non-informative prior probability distribution because information on the priors was not obtainable. All data management and statistical analyses were performed using SAS software and R software version 3.6.2, with the R-INLA package.

## Results

**Fig. 1:**
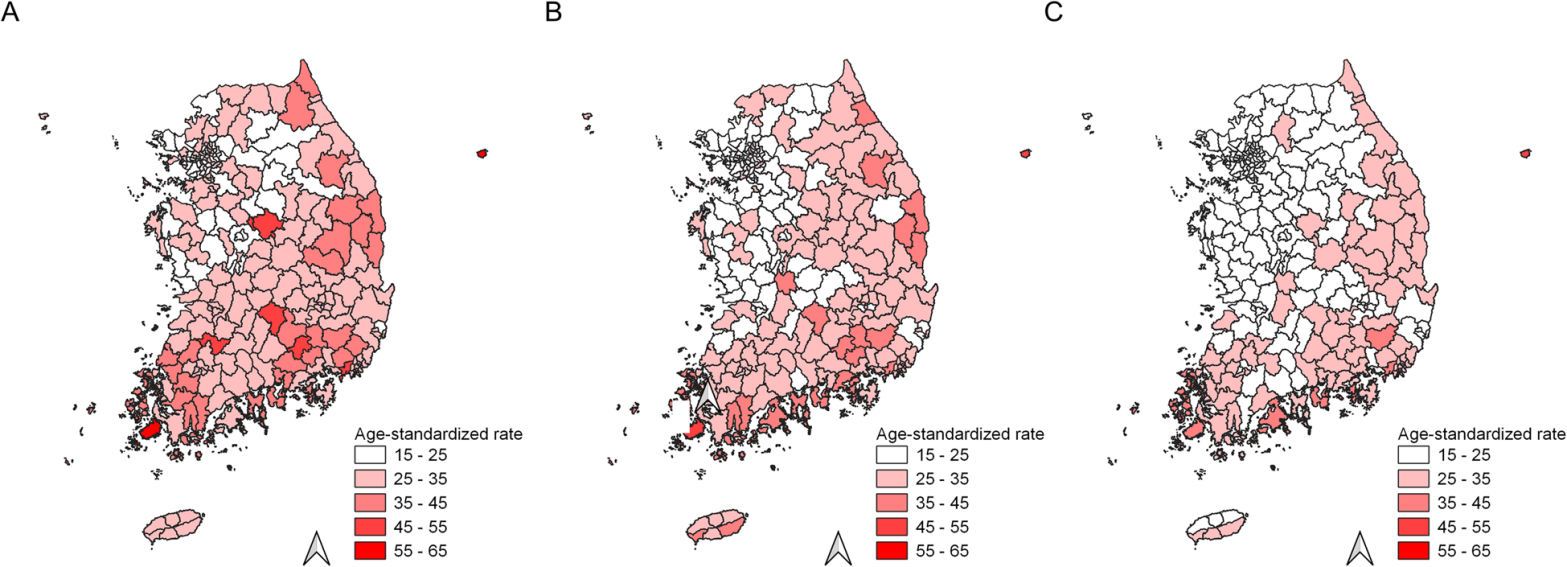
Choropleth maps of age-standardized incidence rates for liver cancer in Korea**. A** Choropleth maps of liver cancer incidence rates from 1999 to 2003, **B** Choropleth maps of liver cancer incidence rates from 2004 to 2008, and **C** Choropleth maps of liver cancer incidence rates from 2009 to 2013

**Fig. 2:**
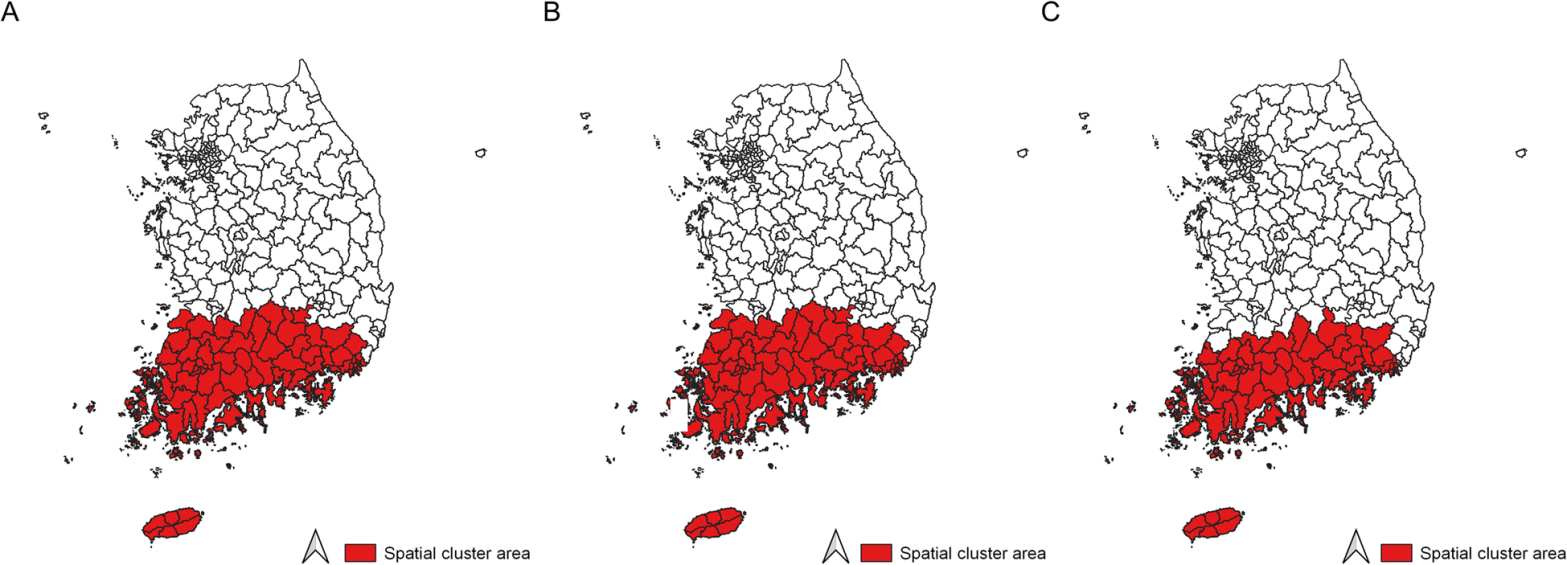
Spatial cluster of liver cancer incidence rates in Korea. **A** Spatial cluster of liver cancer incidence from 1999 to 2003, **B** Spatial cluster of liver cancer incidence from 2004 to 2008, and **C** Spatial cluster of liver cancer incidence from 2009 to 2013

Choropleth maps for liver cancer incidence in Korea from three periods (1999–2003, 2004–2008, and 2009–2013) are presented in Fig. 1. From 1999 to 2013, a steady decrease in liver cancer incidence rate was observed (ASR, 27.5/100,000 for 1999–2003; ASR, 25.8/100,000 for 2004–2008; and ASR, 22.7/100,000 for 2009–2013, respectively) (Table 1). Liver cancer incidence rates in the Gangwon province (northeast province), South Gyeongsang province (southeast province), and South Jeolla province (southwest province) were higher than those in other regions. However, a significant spatial cluster for liver cancer incidence was observed only in the southeast and southwest regions during all three periods (Fig. 2). The incidence rates of liver cancer in the region inside and outside the cluster are estimated to be 34.4 and 27.4 per 100,000 in 1999–2003, 31.9 and 25.6 per 100,000 in 2004–2008, and 28.7 and 22.6 per 100,000 in 2009–2013, respectively (Table 1).

**Table 1:** Age-standardized incidence rates for liver and gallbladder cancer in 1999–2003, 2004–2008, and 2009–2013 in Korea

Period	Liver cancer					Gallbladder cancer				
	Inside the cluster		Outside the cluster		Total area	Inside the cluster		Outside the cluster		Total area
	N of districts	Incidence rate^a^	N of districts	Incidence rate^a^	Incidence rate^a^	N of districts	Incidence rate^a^	N of districts	Incidence rate^a^	Incidence rate^a^
1999–2003	74	34.4	173	27.4	27.5	75	8.4	172	6.1	6.7
2004–2008	74	31.9	173	25.6	25.8	53	9.0	194	6.4	6.9
2009–2013	74	28.7	173	22.6	22.7	74	8.0	173	6.1	6.5

*Abbreviation*: *N* Number

^a^Age standardized incidence rate (cases per 100,000)

We compared the regional characteristics between regions inside and outside the spatial cluster (Supplementary Table 2). The prevalences of hepatitis B infection, heavy alcohol consumption, receiving liver cancer screenings, who were married people and living with their spouses, visiting a public health center at least once within the last year, and people who were diagnosed with gastroduodenal ulcers in the spatial cluster region with high liver cancer incidence was significantly higher than those of regions outside the cluster (all *p*-values < 0.05). In contrast, the regional prevalence of high household income (≥500,000 won), a higher percentage of males in the population, and obesity was lower in the cluster region with high liver cancer incidence than in the areas outside the clustered region (all *p*-values < 0.05). The proportion of Sigungu districts located within 10 km of the national rivers near the basin that showed high *C. sinensis* infection prevalence was larger in the spatially clustered region (60.8%) than outside the clustered region (27.8%) (*p* < 0.01).

Furthermore, we evaluated the association between the characteristics of each Sigungu district and the liver cancer incidence rate based on the Bayesian spatial regression model to take the spatial dependence in the neighborhood into account (Table 2). The regional liver cancer incidence rate was negatively associated with the regional prevalence of high household income (coefficient, − 0.10; 95% CI, − 0.18, − 0.02) and people living with their spouses (coefficient, − 0.14; 95% CI, − 0.25 to − 0.03). A 1% increase in the regional prevalence of patients with hepatitis B infections was significantly linked to an increase of 0.87 per 100,000 (95% CI, 0.29 to 1.44) in the liver cancer incidence rate. A high regional proportion of people who had undergone liver cancer screening at least once within the last year was significantly related to elevated regional liver cancer incidence (coefficient, 0.06; 95% CI, 0.00 to 0.12.)

**Table 2:** Association between regional characteristics and age-standardized incidence rates for liver cancer in 2009–2013 in Korea

Prevalence of characteristics (%)	Median	Range	N (%)	Coefficient	Lower 95% CI	Upper 95% CI
Basic livelihood security recipient	3.7	0.3–12.8	–	0.09	−0.20	0.37
House income ≥500,000 won	10.6	2.0–51.9	–	− 0.10	− 0.18	− 0.02
Living with a partner	65.1	50.7–72.3	–	− 0.14	− 0.25	− 0.03
Heavy alcohol consumption^a^	6.5	2.7–12.0	–	0.05	−0.27	0.37
Heavy smoking^b^	1.8	0.1–4.4	–	0.15	− 0.05	0.34
Moderate physical activity^c^	5.5	0.6–18.8	–	0.06	−0.10	0.21
Walking^d^	17.0	2.8–31.7	–	−0.06	−0.15	0.03
Hepatitis B diagnosis	1.8	0.2–4.3	–	0.87	0.29	1.44
Gastroduodenal ulcer diagnosis	2.5	0.3–11.2	–	0.13	−0.14	0.40
Diabetes mellitus diagnosis	6.3	2.2–9.9	–	0.16	−0.22	0.54
Health checkup^e^	58.8	49.3–70.7	–	−0.04	−0.18	0.10
Liver cancer screening examinee within the last 2 years	20.9	7.7–61.2	–	0.06	0.00	0.12
Public health center usage^f^	27.2	7.7–82.4	–	0.02	−0.05	0.08
Distance from rivers < 10 km^g^	–	–	93 (37.7)	−0.86	−1.85	0.13

*Abbreviations*: *N* Number, *CI* Credible interval

^a^Alcohol consumption occasions ≥2–3 times per week

^b^Smoking ≥20 cigarettes per day

^c^Moderate and vigorous physical activity ≥4 times per week

^d^Walking physical activity ≥4 times per week

^e^Receiving health checkup at least once within the last 2 years

^f^Visiting public health center at least once within the last year

^g^Distance from rivers located in regions with high *C. sinensis* infection prevalence to the centroid of each district < 10 km

No significant decrease in gallbladder cancer incidence rates in 1999–2013 was observed in Korea (ASR, 6.7/100,000 for 1999–2003; ASR, 6.9/100,000 for 2004–2008; and ASR, 6.5/100,000 for 2009–2013, respectively) (Table 1) as reported by the National Cancer Center. Higher gallbladder incidence rates in 2009–2013 than those in 1999–2003 were observed in several Sigungu districts.

**Fig. 3:**
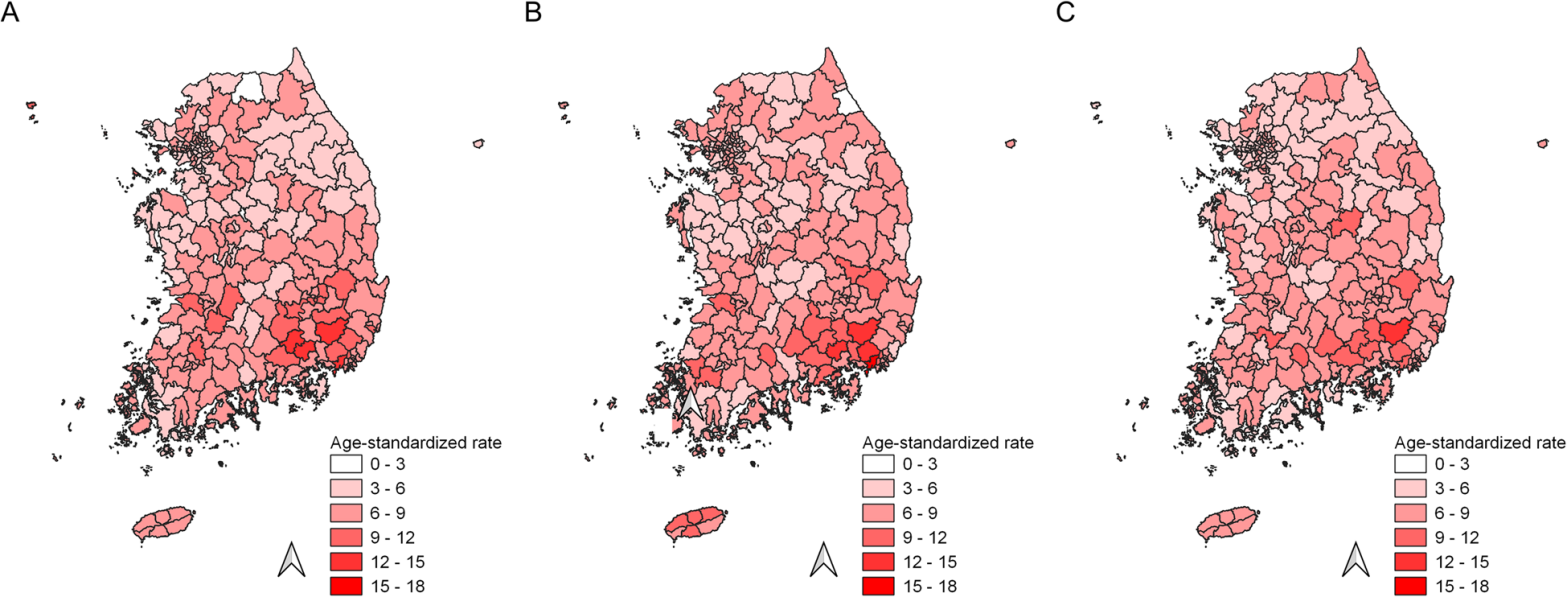
Choropleth maps of age-standardized incidence rates for gallbladder cancer in Korea. **A** Choropleth maps of gallbladder cancer incidence from 1999 to 2003, **B** Choropleth maps of gallbladder cancer incidence from 2004 to 2008, and **C** Choropleth maps of gallbladder cancer incidence from 2009 to 2013

**Fig. 4:**
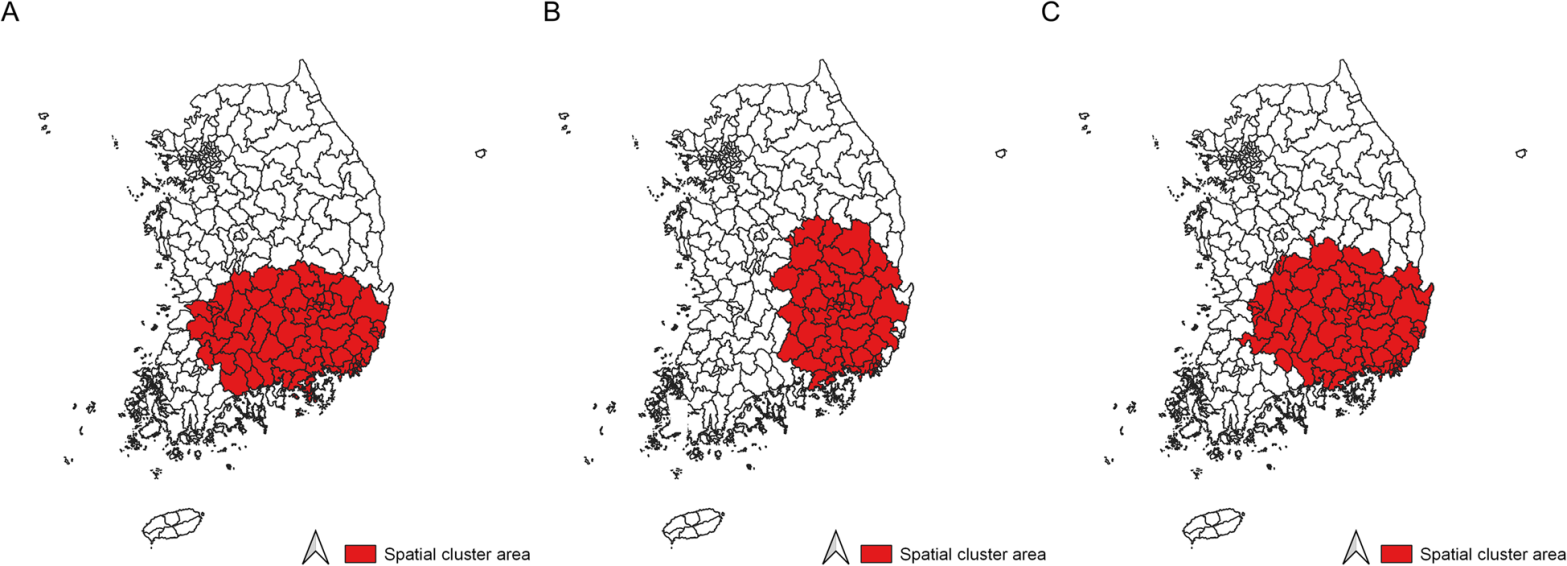
Spatial cluster of gallbladder cancer incidence rates in Korea. **A** Spatial cluster of gallbladder cancer incidence rates from 1999 to 2003, **B** Spatial cluster of gallbladder cancer incidence rates from 2004 to 2008, and **C** Spatial cluster of gallbladder cancer incidence rates from 2009 to 2013

Higher incidence rates for gallbladder cancer were mainly observed in South Gyeongsang province than in other regions in all three periods from 1999 to 2013 (Fig. 3). Spatial clusters for gallbladder cancer incidence consistently included regions within the South Gyeongsang province from 1999 to 2013 (Fig. 4). For the periods 1999–2003 and 2009–2013, the spatial cluster for gallbladder cancer also comprised several Sigungu districts within the North and South Jeolla provinces. Estimated gallbladder cancer incidence rates of regions inside and outside the cluster were 8.4 and 6.1 per 100,000 in 1999–2003, 9.0 and 6.4 per 100,000 in 2004–2008, and 8.0 and 6.1 per 100,000 in 2009–2013, respectively (Table 1).

Regions within the spatial cluster with high gallbladder cancer incidence rates had low prevalences of high household income, living with a partner, male, moderate physical activity, and obesity (all *p*-values < 0.05) (Supplementary Table 3).

Spatial analysis was performed to identify the determinants of regional variation in gallbladder cancer incidence (Table 3). High regional prevalence of high household income (≥500,000 won) was correlated with a decreased gallbladder cancer incidence rate (coefficient, − 0.03; 95% CI, − 0.05 to 0). The region near the national rivers, which is known for a high incidence of *C. sinensis* infection, had a high gallbladder cancer incidence rate (coefficient, 0.55; 95% CI, 0.14 to 0.96).

**Table 3:** Association between regional characteristics and age-standardized incidence rates for gallbladder cancer in 2009–2013 in Korea

Prevalence of characteristics (%)	Median	Range	N (%)	Coefficient	Lower 95% CI	Upper 95% CI
Basic livelihood security recipient	3.7	0.3–12.8	–	0.04	−0.07	0.14
House income ≥500,000 won	10.6	2.0–51.9	–	−0.03	−0.05	0.00
Living with a partner	65.1	50.7–72.3	–	−0.03	−0.07	0.01
Heavy alcohol consumption^a^	6.5	2.7–12.0	–	−0.06	−0.18	0.05
Heavy smoking^b^	1.8	0.1–4.4	–	0.00	−0.07	0.07
Moderate physical activity^c^	5.5	0.6–18.8	–	−0.04	−0.09	0.02
Walking^d^	17.0	2.8–31.7	–	−0.00	−0.03	0.03
Hepatitis B diagnosis	1.8	0.2–4.3	–	0.12	−0.08	0.33
Gastroduodenal ulcer diagnosis	2.5	0.3–11.2	–	−0.07	−0.17	0.02
Diabetes mellitus diagnosis	6.3	2.2–9.9	–	−0.11	−0.24	0.03
Health checkup^e^	58.8	49.3–70.7	–	0.03	−0.02	0.08
Liver cancer screening examinee within the last 2 years	20.9	7.7–61.2	–	0.00	−0.02	0.02
Public health center usage^f^	27.2	7.7–82.4	–	0.01	−0.02	0.03
Distance from rivers < 10 km^g^	–	–	93 (37.7)	0.55	0.14	0.96

*N* Number, *CI* Credible interval

^a^Alcohol consumption occasions ≥2–3 times per week

^b^Smoking ≥20 cigarettes per day

^c^Moderate and vigorous physical activity ≥4 times per week

^d^Walking physical activity ≥4 times per week

^e^Receiving health checkup at least once within the last 2 years

^f^Visiting public health center at least once within the last year

^g^Distance from rivers located in regions with high *C. sinensis* infection prevalence to the centroid of each district < 10 km

## Discussion

In this study, we demonstrated geographical variations in the incidence of liver and gallbladder cancer in 1999–2013 in Korea. Significant spatial clusters for liver cancer incidence were identified in the South Jeolla and South Gyeongsang provinces throughout the study period. For gallbladder cancer, spatial clusters included almost the entire area in the South Gyeongsang province and several Sigungu districts in the North and South Jeolla provinces.

Furthermore, we examined the determinants of geographical variation in the incidence of liver and gallbladder cancer. As hypothesized, the high regional prevalence of hepatitis B infection was significantly correlated with an increase in the liver cancer incidence rate. Additionally, the districts with high proportions of high household income and people living with their spouses tended to have low liver cancer incidence rates. The regional proportion of people receiving liver cancer screening within the last 2 years was positively associated with the regional incidence rate of liver cancer. The high regional prevalence of high household income was significantly associated with a low gallbladder cancer incidence rate. We also found that the high gallbladder cancer incidence rate was linked to living in the Sigungu districts near rivers located in areas with a high prevalence of *C. sinensis* infection.

Other studies have aimed to assess the geographic pattern of liver cancer incidence and to identify factors linked to regional variation in liver cancer incidence rates [19–21], similar to this study. However, one of the abovementioned studies was limited to finding spatial clusters for liver cancer and simply comparing the sociodemographic characteristics between the clustered regions and the rest of the state [19]. Other studies were similar to the present study in that they tried to identify factors that could explain regional variation based on spatial analysis. In New York City, it was found that the spatial distribution of liver cancer incidence can be linked to hepatitis B and C incidence and poverty [21]. However, major risk factors for liver cancer, such as smoking or alcohol consumption, were not considered in the analysis. Another study aimed to explain regional differences in liver cancer incidence in the province of Ontario, Canada based on a spatial model including potential determinants ranging from age, sex, geographic location, immigration, smoking, alcohol consumption, physical activity, obesity, fruit and vegetable preference, diabetes, education level, and income [20]. This study differs from our study in that information on hepatitis B or C infection was not available and only the proportion of immigrants could account for the geographical variation in the incidence of liver cancer.

Spatial analysis of the incidence of gallbladder cancer has rarely been conducted, and only a few studies have been performed to explore the regional distribution of gallbladder cancer mortality rates and their associated factors [22–24]. Moreover, in the case of the above studies, the only factor expected to affect gallbladder cancer mortality was living near industrial areas, or even if potential determinants including typhoid fever, ethnicity, socioeconomic status, and cholecystectomy were considered, there was a limitation in their spatial analyses, namely that autocorrelation was not considered.

The findings on the determinants of geographical variation in liver cancer incidence are plausible. We found that regional liver cancer incidence was positively correlated with hepatitis B infection, which was defined as a class I carcinogen by the International Agency for Research on Cancer [25]. Based on the existing reports of the regional variation in the incidence of hepatitis B in Korea [7], the significant association between regional variation in hepatitis and liver cancer incidence is sufficiently explanatory.

This study also supports the hypothesis that some part of the spatial variability in liver cancer incidence is due to household income and marital status. Several studies have reported that people with a low socioeconomic status have an increased risk for liver cancer [26, 27] may be due to poor health behaviors including alcohol consumption [28], smoking [29], and high hepatitis B [30] and C incidence rates [31]. However, regional variations in alcohol consumption and smoking could not be attributed to the spatial variation in liver cancer incidence in this study, and household income was still correlated with the regional liver cancer incidence rate even after adjusting for the impact of alcohol consumption, smoking, and hepatitis B infection. These results suggest that factors other than alcohol consumption, smoking, and hepatitis B result in regional variability in liver cancer incidence in Korea.

Studies on the impact of marital status on health behavior have shown that people living with a partner are less likely to drink alcohol, smoke, and be obese than people living without a partner [32]. Furthermore, it was reported that unmarried people are more likely to be unaware of the fact that they have a hepatitis B infection; as a result, there is a possibility that they are not able to take appropriate measures to prevent liver cancer development [33].

Part of the regional variation in liver cancer incidence could be explained by regional differences in liver cancer screening in this study. This may be derived from that low stage or small tumors could be detected more frequently in areas with a high liver cancer screening prevalence.

Household income and distance from rivers near districts with high liver fluke infection prevalence were significantly associated with regional variability in gallbladder cancer incidence in this study. The negative correlation between regional household income and gallbladder cancer incidence rates can be interpreted as a significant association between socioeconomic status and health behaviors and conditions, including obesity [34], smoking [29], and alcohol consumption [28] which are known risk factors for gallbladder cancer.

It is scientifically plausible that the districts located within 10 km from the national rivers near areas with high *C. sinensis* infection prevalence have a higher incidence rate of gallbladder cancer than other districts that are not. *C. sinensis* is a well-known risk factor for gallbladder cancer [3]. There have been reports on regional variation in *C. sinensis* infection prevalence in Korea [8, 9]; therefore, the possibility of spatial variability in gallbladder cancer incidence in Korea due to *C. sinensis* infection has been considered. Additionally, there was a recent study on the regional variation in gallbladder cancer incidence rates across 16 cities and provinces in Korea [35]. The authors suggested *C. sinensis* as a factor influencing regional variation in gallbladder cancer incidence in the same study; however, this has a limitation in that it was just a suggestion based on a literature review, not an inference based on an analysis. Although information on the regional prevalence of *C. sinensis* infection could not be obtained in this study, for the first time, the association between regional variations in gallbladder cancer and *C. sinensis* infection prevalence in Korea was more objectively presented because the distance from the four national rivers located in the region with high *C. sinensis* infection was included as a proxy variable in the spatial analysis.

Several limitations should be considered when interpreting the results of this study. First, this study was performed based on an ecological study design; thus, the establishment of causal relationships based on our results requires caution. Second, we used district-level aggregated information on disease outcome and its potential risk factors; therefore, loss of information may arise compared to the use of individual-level data to assess regional variation in disease outcome and its determinants. Third, the latency period between carcinogen exposure and cancer development was not considered in this study. However, considering that we used covariate information collected before cancer incidence information and regional characteristics such as sociodemographic status, health behaviors, and the fact that health care utilization may not change dramatically in a short period, lack of consideration of the latency period might not have a significant impact on the association between regional liver and gallbladder cancer incidence and its determinants. Fourth, information on major risk factors for liver and gallbladder cancer, including *C. sinensis* and gallstones, is lacking. Therefore, we used the distance between major rivers known to be near areas with high *C. sinensis* infection and the centroid of each Sigungu district as a proxy variable for the regional characteristics of *C. sinensis* infection. Fifth, we did not consider the stage of cancer in the analysis. The positive correlation between the liver cancer screening prevalence and the regional liver cancer incidence rate might be interpreted as the early detection of cancer. If the liver cancer stage could be determined, we might be able to provide evidence on whether the significant association between the regional prevalence of liver cancer screening and regional liver cancer incidence rate is the result of early detection or because there are many residents with symptoms in the region with high liver cancer screening prevalence.

Despite the limitations above, this is the first national study to identify the spatial clusters of liver and gallbladder cancer incidence rates across Korea and to identify the determinants of regional variability based on spatial analysis. Spatial autocorrelation of disease outcomes and potential determinants was considered by assigning neighbor spatial weights to the spatial model. Therefore, estimations of the association between regional characteristics and regional liver and gallbladder cancer incidence would be less biased than estimations obtained from a simple regression model.

## Conclusions

This is an exploratory study that can provide a better understanding of the spatial epidemiology of liver and gallbladder cancer and provide a basis for cancer prevention and control by assessing the determinants of regional distribution of liver and gallbladder cancer. We found regional variability in liver and gallbladder cancer incidence rates in Korea. The determinants of liver cancer were socioeconomic status, hepatitis B infection incidence, and regular liver cancer screening, while household income and the distance between each district and rivers near the region with high *C. sinensis* infection prevalence were determinants of gallbladder cancer.

The results of this study provide a basis for the necessity of identifying modifiable risk factors of liver and gallbladder cancer such as hepatitis B and *C. sinensis* infections in regions with high incidence rates of liver and gallbladder cancer and implementing appropriate medical resource distribution. If the potential determinants of regional variation in liver and gallbladder cancers are more diverse and analyses based on individual-level data are performed in subsequent studies, it is expected that a more effective solution can be found to resolve the regional inequity of liver and gallbladder cancers and to lower the incidence of cancer.

## Supplementary Information

**Additional file 1.**

## Data Availability

The datasets analysed during the current study are available in the Korean Statistical Information Service (KOSIS), [https://kosis.kr/statisticsList/statisticsListIndex.do?vwcd=MT_ZTITLE&menuId= M_01_01&outLink = Y&entrType = #content-group], and Korea Community Health Survey (KCHS) data repository, [https://chs.kdca.go.kr/chs/rdr/rdrInfoProcessMain.do].

## Abbreviations

ASR: Age-standardized incidence rates
*C. sinensis*: *Clonorchis sinensis*
KCHS: Korea Community Health Survey
