# Supplementary material for: Spatial epidemiologic analysis of the liver cancer and gallbladder cancer incidence and its determinants in South Korea
Source: BMC Public Health. 2021 Nov 14;21:2090. doi: 10.1186/s12889-021-12184-8 (PMC8590754; doi:10.1186/s12889-021-12184-8)
Supplement: Supplementary file 1 — Additional file 1. [file 12889_2021_12184_MOESM1_ESM.docx]

Supplementary Table 1. List of covariates considered in analyses to identify determinants of spatial clusters of liver and gallbladder cancer

| Variables | Min | Median | Max | Year |
| --- | --- | --- | --- | --- |
| Male (%) | 46.6 | 49.6 | 55.3 | 2009 |
| Living with a partner (%) | 50.7 | 65.1 | 72.3 | 2009 |
| Education ≥12 years (%) | 34.6 | 67.5 | 93.3 | 2009 |
| Basic livelihood security recipient (%) | 0.3 | 3.7 | 12.8 | 2009 |
| House income ≥ 500,000 won (%) | 2.0 | 10.6 | 51.9 | 2009 |
| Cancer screening examinee within 2 years (%) | 24.9 | 45.9 | 65.1 | 2010 |
| Liver cancer screening examinee within 2 years (%) | 7.7 | 20.9 | 61.2 | 2010 |
| Diabetes mellitus diagnosis (%) | 2.2 | 6.3 | 9.9 | 2009 |
| Health checkup within 2 years (%) | 49.3 | 58.8 | 70.7 | 2009 |
| Public health center usage within 1 year (%) | 7.7 | 27.2 | 82.4 | 2009 |
| Heavy alcohol consumption^1^ (%) | 2.7 | 6.5 | 12.0 | 2009 |
| Heavy smoking^2^ (%) | 10.6 | 19.3 | 27.3 | 2009 |
| Obesity^3^ (%) | 14.6 | 22.1 | 30.7 | 2009 |
| Moderate intensity or vigorous physical activity^4^ (%) | 0.6 | 5.5 | 18.8 | 2009 |
| Walking physical activity^5^ (%) | 2.8 | 17.0 | 31.7 | 2009 |
| Hepatitis B diagnosis (%) | 0.2 | 1.8 | 4.3 | 2009 |
| Gastroduodenal ulcer diagnosis (%) | 0.3 | 2.5 | 11.2 | 2009 |
| Distance from river to centroid of district (km)^6^ | 0 | 20.4 | 195.3 | 2004 |

Min, Minimum value; Max, Maximum value

1. Alcohol consumption occasions ≥2-3 times per week
2. Smoking ≥20 cigarettes per day
3. Body mass index ≥25.0 kg/m^2^
4. Moderate intensity physical activity ≥4 times per week
5. Walking physical activity ≥4 times per week
6. Distance from rivers located in regions with high C. sinensis infection prevalence to the centroid of each district < 10 km

Supplementary Table 2. Comparison of characteristics between regions inside and outside of the liver cancer cluster in the period–2009-2013 in Korea

| Prevalence of characteristics (%) | Inside the cluster | Outside the cluster | *p*-value |
| --- | --- | --- | --- |
|  | Median (Range) | Median (Range) |  |
| Basic livelihood security recipient | 5.2 (1.5-11.3) | 3.2 (0.3-12.8) | <0.01 |
| House income ≥ 500,000 won | 8.3 (2.1-23.6) | 11.9 (2.0-51.9) | <0.01 |
| Living with a partner | 65.6 (50.7-72.2) | 63.5 (51.2-72.3) | 0.01 |
| Male | 49.0 (46.6-52.4) | 49.8 (46.6-55.3) | <0.01 |
| Heavy alcohol consumption^1^ | 6.8 (2.7-11.8) | 6.3 (2.9-12.0) | 0.01 |
| Heavy smoking^2^ | 19.4 (14.4-27.3) | 19.3 (10.6-26.8) | 0.68 |
| Moderate physical activity^3^ | 5.4 (1.0-16.7) | 5.6 (0.6-18.8) | 0.40 |
| Obesity^4^ | 20.7 (16.5-25.6) | 23.3 (14.6-30.7) | <0.01 |
| Hepatitis B diagnosis | 2.2 (0.2-4.3) | 1.8 (0.2-3.8) | <0.01 |
| Liver cancer screening examinee within 2 years | 23.7 (11.0-61.2) | 20.2 (7.7-50.8) | <0.01 |
| Gastroduodenal ulcer diagnosis | 2.9 (0.4-8.1) | 2.4 (0.3-11.2) | 0.03 |
| Public health center usage^5^ | 34.5 (13.1-71.7) | 23.0 (7.7-82.4) | <0.01 |
|  | N (%) | N (%) |  |
| Distance from river <10 km | 45 (60.8) | 48 (27.8) | <0.01 |

N, Number

1. Alcohol consumption occasions ≥2-3 times per week
2. Smoking ≥20 cigarettes per day
3. Moderate and vigorous physical activity ≥4 times per week
4. Body mass index ≥25.0 kg/m^2^
5. Visiting public health center at least once within the last year

Supplementary Table 3. Comparison of characteristics between regions inside and outside of the gallbladder cancer cluster in the period–2009-2013 in Korea

| Prevalence of characteristics (%) | Inside the cluster | Outside the cluster | *p*-value |
| --- | --- | --- | --- |
|  | Median (Range) | Median (Range) |  |
| Basic livelihood security recipient | 4.1 (1.0-11.3) | 3.5 (0.3-12.8) | 0.36 |
| House income ≥ 500,000 won | 8.8 (2.3-36.5) | 11.8 (2.0-51.9) | 0.04 |
| Living with a partner | 63.8 (50.7-72.3) | 65.6 (51.7-72.0) | 0.02 |
| Male | 49.2 (47.0-52.4) | 49.7 (46.6-55.3) | 0.01 |
| Heavy alcohol consumption^1^ | 6.5 (2.7-11.0) | 6.5 (2.9-12.0) | 0.92 |
| Heavy smoking^2^ | 19.7 (14.1-24.9) | 19.1 (10.6-27.3) | 0.50 |
| Moderate physical activity^3^ | 4.8 (0.6-12.7) | 5.8 (0.7-18.8) | 0.01 |
| Obesity^4^ | 20.7 (15.5-26.2) | 23.0 (14.6-30.7) | <0.01 |
| Hepatitis B diagnosis | 1.9 (0.2-4.3) | 1.8 (0.2-4.0) | 0.67 |
| Liver cancer screening examinee within 2 years | 22.1 (11.0-50.8) | 20.5 (7.7-61.2) | 0.61 |
| Gastroduodenal ulcer diagnosis | 2.8 (0.4-8.1) | 2.4 (0.3-11.2) | 0.33 |
| Public health center usage^5^ | 25.8 (11.6-71.7) | 27.7 (7.7-82.4) | 0.58 |
|  | N (%) | N (%) |  |
| Distance from river <10km | 58 (78.4) | 35 (20.2) | <0.01 |

N, Number

1. Alcohol consumption occasions ≥2-3 times per week
2. Smoking ≥20 cigarettes per day
3. Moderate and vigorous physical activity ≥4 times per week
4. Body mass index ≥25.0 kg/m^2^
5. Visiting public health center at least once within the last year
